# Prevalence of overweight and obesity among primary school-aged children in Jiangsu Province, China, 2014-2017

**DOI:** 10.1371/journal.pone.0202681

**Published:** 2018-08-23

**Authors:** Xiyan Zhang, Fengyun Zhang, Jie Yang, Wenyi Yang, Weina Liu, Liuwei Gao, Zhihang Peng, Yan Wang

**Affiliations:** 1 Department of Child and Adolescent Health Promotion, Jiangsu Provincial Center for Disease Control and Prevention, Nanjing, China; 2 Public Health Research Institute of Jiangsu Province, Nanjing, China; 3 Department of biostatistics, School of public health, Nanjing medical university, Nanjing, China; Soochow University Medical College, CHINA

## Abstract

**Background:**

Data was limited on prevalence of overweight and obesity among primary school-aged children in Jiangsu Province. We aimed to present the current situation of obesity in Jiangsu Province and explore the relationship between obesity and other common diseases in children.

**Methods:**

Physical examination among children aged 7 to 14 years in Jiangsu Province was conducted since 2014, and more than one third primary schools were covered annually. The physical measurements included body height, weight, blood pressure, vision, sex, age, and so on.

**Results:**

The prevalence of overweight and obesity among primary school children was 15.2% (18.7% for male students and 11.0% for female students), and 11.7% (14.5% for male students and 8.2% for female students) respectively. Obesity/overweight prevalence varied by regions. Among them the lowest prevalence was found in the southern region of Jiangsu Province, where residents had the highest average income level. Obesity group had elevated blood pressure comparing with the normal group, and obesity group especially in the male children aged 7 to 12 years had a higher prevalence of uncorrected visual acuity (UCVA) than that of normal group.

**Conclusion:**

This study found that obesity/overweight prevalence differed by sex, age, and regions in Jiangsu Province. In addition, obese children were closely associated with other common disease. Further studies are needed to explore the basis of biological and statistical theories.

## Introduction

The prevalence of obesity among children and adolescents has increased dramatically during the past decades all over the world[1, 2]. Meanwhile, childhood obesity is a multisystem disease with potentially devastating results. It can develop serious medical and psychosocial complications and greatly increase the risk of morbidity and mortality in adults[3].

It has been reported that about 30% to 50% of childhood obesity will continue to become obese adults[4], and obesity may vary by regions, population, climate factors, economic levels, and so on[5, 6]. Marie Ng et found that the prevalence of obesity has increased substantially in children in both developed and developing countries[1]. In China, the prevalence of childhood obesity has gradually increased to the point where it is now similar to developed countries[7, 8]. Several studies were also conducted in China. Ji CY collected data from National Surveys on Chinese Students’ Constitution and Health which were carried in 1985, 1991, 1995 and 2000 suggesting that the prevalence of both obesity and overweight remained relatively low[8]. In adults, however, prevalence of obesity increased by 97.2% from 1992 to 2002[9]. Xi B et also reported that prevalence of obesity increased 1.68 times from 1993 to 2009 in Chinese adults[10].

In addition, childhood obesity significantly contributes to related adverse health problems, such as dyslipidemia, hypertension, cardiovascular disease, insulin resistance or diabetes, asthma, fatty liver disease, and psychosocial complications[11–13].

Located in the eastern China, Jiangsu Province is one of the developed regions in China. We conducted a successive surveillance among primary school children here since 2014, More than 100,000 students were included in the study annually which covers over 40% area of the province. The purpose of this study was to estimate the prevalence of childhood overweight and obesity among primary school children in Jiangsu Province, and observe the trend of body mass index (BMI) from 2014–2015 to 2016–2017 academic season. We also explored relationships between obesity and other common diseases in student to further understand the harmful effects that obesity may bring about.

## Materials and methods

### Study sites

This study was conducted in Jiangsu Province, which divided into 13 cities consisting of 97 districts or counties. We selected 40districts or counties which were taken up 41.2% regions of this province since 2014, and fifty-three primary schools were chosen into our study with average108, 331 students annually included. (S1 Fig)

### Data collection

Students in all schools were included in cluster sampling and received physical examination from 2014 to 2017. Weight and height were measured after students had removed shoes and clothes. Height (measured to nearest 0.1 cm) and weight (measured to the nearest 0.1 kg) were measured using standardized equipment and procedures according to *Health check list for primary and junior school students* (*GB 16134–2011*). BMI was calculated as weight in kilograms divided by height in meters squared. Blood pressure and defects of vision were also measured in this study. Uncorrected visual acuity (UCVA) was measured for the right eye, followed by the left eye, with an E Standard Logarithm Vision Acuity Chart (GB11533-2011) in 5-grade notation, with illumination of the chart around 500 lx. The 5-grade notation was obtained using the formula: L = 5-LogMAR. The children were asked to indicate the direction of the E optotype within 5 seconds. They were asked to start with the fourth line, if optotypes of the line were correctly described, otherwise the previous line was continued. When the children falsely described at least 1 character in the 5.1–5.3 line, or at least 2 characters in the 4.6–5.0 line, or at least 3 characters in the 4.0–4.5line, visual acuity was recorded as the value of the previous line. Visual acuity less than 5.0 in 5 graded is defined as UCVA[14]. All students also filled in a simple questionnaire to provide basic demographic information such as name, sex, age and so on.

### Definition for overweight and obesity

Obesity is defined by BMI in this study, and We used unified classification criteria for overweight, obesity and BMI screening among children and adolescents by Working Group on Obesity in China (WGOC)[15] (S1 Table).

### Statistical analysis

Descriptive statistics were used to summarize variables concerning characteristics of primary school students, and the prevalence of overweight and obesity were presented as the prevalence and 95% confidence interval(CI) of the prevalence. Continuous variables like blood pressure were presented as the mean with standard deviation(SD) of the mean. Student *t* tests were used for comparisons of 2-group with continuous variables, and Chi-square tests were performed for comparing two proportions of UCVA. Poisson regression analysis was performed and adjusted risk ratio values were computed to assess the relationship between obesity and other common childhood disease[16–18]. Data was analyzed by SPSS 20.0 and SAS9.2 software, and figures were drawn by ArcGis10.0 and office software.

### Ethics statement

The study protocol was approved by the Institutional Review Board of Ethics committee of Jiangsu Province. Ministry of education published an Official Notice of *Implementation Plan of National Student Physical Health Monitoring Network Work* since 2002, which was belonging as part of nine years of compulsory education welfare(Specific content could be acquired by landing on the URL: http://www.moe.gov.cn/jyb_xxgk/gk_gbgg/moe_0/moe_8/moe_25/tnull_285.html). According to this Official Notice, Education Department of Jiangsu Province conducted physical examination for students every year since 2005. During the period of student’s physical examination, the Centers for Disease Control and prevention would be invited to provide technical guidance. Therefore, acquisition of informed consent is mainly organized by the Education Department and the Health Department will require school to keep a written consent in the name of school as an indicator of annual performance check.

Detailed implementation process can be described as follows: At the beginning of each semester schools will hold parent-teacher conference, and every student’s guardian would be consulted by teachers. If parents do not intend to attend the examination, they will tell the teacher verbally, and the other students will be regarded as oral informed consent. Certainly, if it involves extraction of biological materials such as blood, pleural effusion, CSF sampling, there will be a written consent.

## Results

In our study 108,331 observations were enrolled annually and a total of 324,993 observations were included since 2014, which were consisting of 177,103 male children and 147,980 female children and the male to female ratio was 1.2. Children aged 7 to 12 years accounted for 95% in primary school and more than half were from south of Jiangsu Province. (Table 1)

**Table 1:** Characteristics of primary school students in Jiangsu Province, 2014–2017.

Characteristic		No.	Percentage (%)
Sex	Male	177,103	54.5
	Female	147,980	45.5
Age	7-	57,631	17.7
	8-	55,800	17.2
	9-	55,486	17.1
	10-	55,527	17.1
	11-	51,126	15.7
	12-	33,170	10.2
	13-	10,031	3.1
	14-	6,222	1.9
Area	South	171,327	52.7
	Middle	67,700	20.8
	North	85,966	26.5
Year	2014–2015	107,677	33.2
	2015–2016	108,316	33.3
	2016–2017	109,000	33.5
Total		324,993	100.0

**Fig 1:**
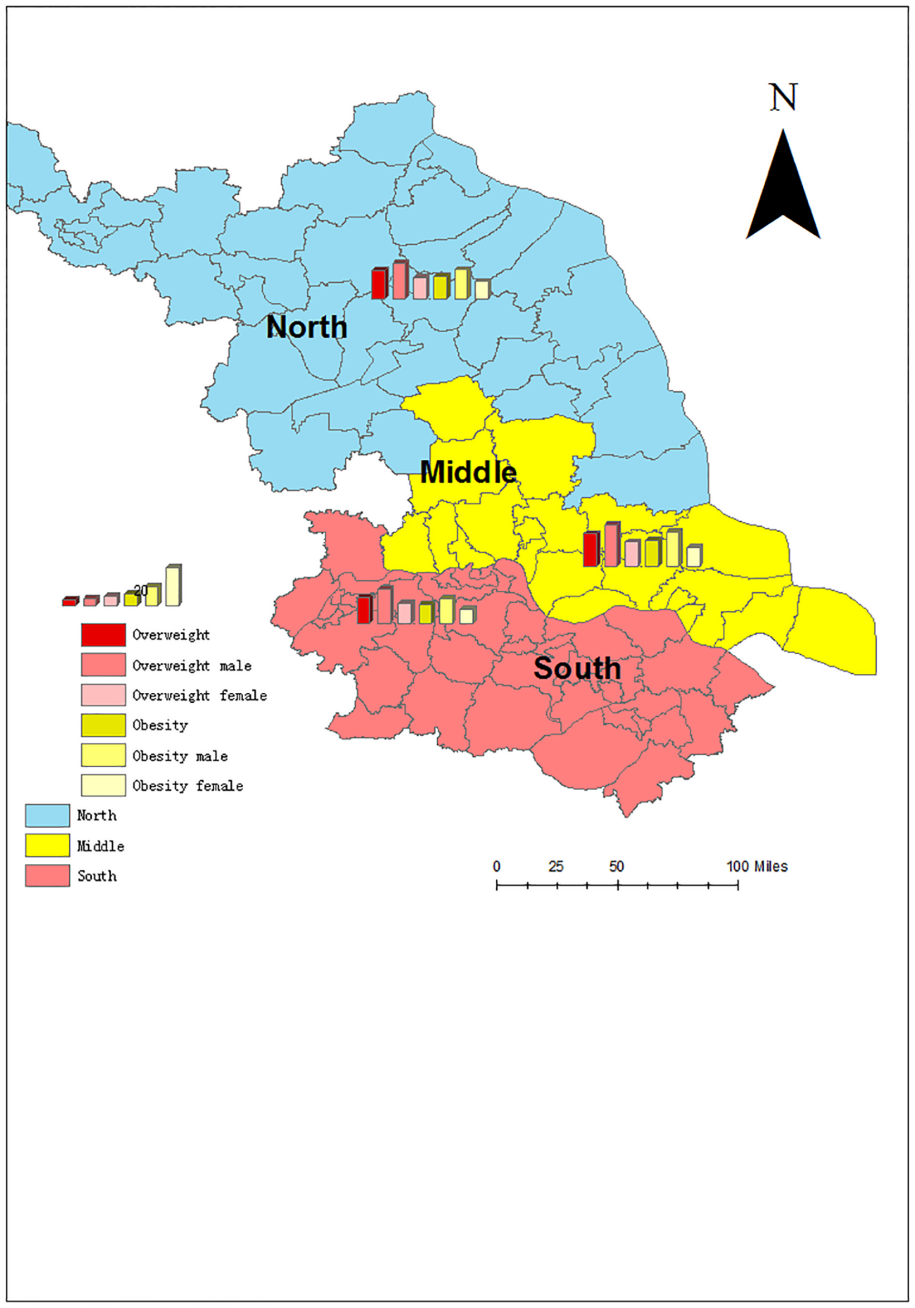
Prevalence of overweight and obesity for students from different regions in Jiangsu Province 2014–2017.

The prevalence of overweight and obesity in the middle of Jiangsu Province ranked at the first place:17.5% for overweight and 14.1% for obesity, and the northern region was at the second place: 15.0% for overweight and 12.3% for obesity. The southern region remained lowest the prevalence of overweight and obesity: 14.2% for overweight and 10.2% for obesity. However, the prevalence of overweight and obesity for male children were always higher than those of female children in the three regions. (P <0.01) (Fig 1).

**Fig 2:**
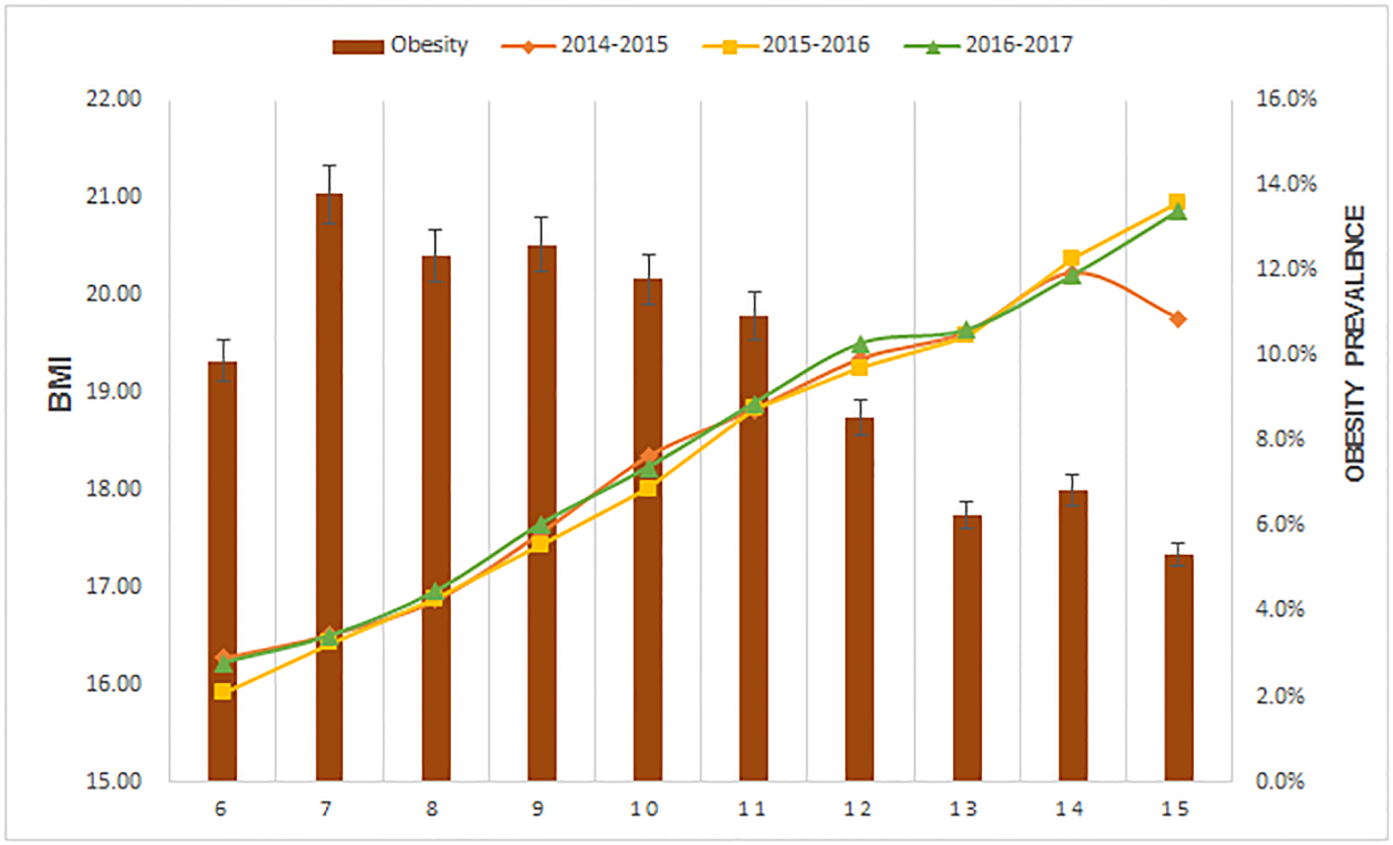
Trend for BMI changes for primary students in Jiangsu Province 2014–2017.

From 2014–2015 to 2016–2017 academic year the average value of BMI maintained stable ranging from 16.5 to 21.0, and the value of BMI increased by age. However, the prevalence of obesity decreased by age ranging from 13.8% to 6.3%. (Fig 2)

The prevalence of combined obesity and overweight among children in primary schools was 26.8%, 95%CI:26.7%-27.0%, which for male students was 33.2%, 95%CI: 33.0%-33.4% and for female students was 19.2%,95%CI:19.0%-19.2%. The prevalence of obesity was 11.7%, 95%CI:11.5%-11.8%, which for male students was 14.5%,95%CI:14.3%-14.7% and for female students was 8.2%, 95%CI:8.1%-8.4%. For children aged 7 years the prevalence of combined overweight and obesity was the highest and for children aged 13 years the prevalence was the lowest. (Table 2)

**Table 2:** Age-specific prevalence for children and adolescents with overweight and obesity for them in Jiangsu Province 2014–2017.

Age	Combined obesity and overweight(95%CI)			Obesity(95%CI)			Overweight (95CI%)		
	Total (%)	Male (%)	Female (%)	Total (%)	Male (%)	Female (%)	Total (%)	Male (%)	Female (%)
7-	28.3(27.9–28.6)	31.8(31.2–32.3)	24.1(23.6–24.6)	13.8(13.5–14.1)	16.2(15.8–16.6)	11.0(10.6–11.3)	14.5(14.2–14.8)	15.6(15.2–16.0)	13.2(12.7–13.6)
8-	26.1(25.8–26.5)	30.6(30.1–31.1)	20.8(20.3–21.3)	12.3(12.1–12.6)	14.8(14.4–15.2)	9.5(9.1–9.8)	13.8(13.5–14.1)	15.9(15.5–16.3)	11.3(10.9–11.7)
9-	27.0(26.7–27.4)	33.6(33.1–34.2)	19.1(18.6–19.6)	12.6(12.3–12.9)	16.0(15.5–16.4)	8.6(8.3–8.9)	14.4(14.1–14.7)	17.7(17.2–18.1)	10.5(10.1–10.9)
10-	27.8(27.4–28.2)	35.7(35.1–36.2)	18.4(17.9–18.9)	11.8(11.5–12.1)	15.1(14.7–15.5)	7.9(7.6–8.2)	16.0(15.7–16.3)	20.6(20.1–21.1)	10.5(10.1–10.9)
11-	27.3(27.0–27.7)	36.6(36.0–37.2)	16.3(15.8–16.7)	10.9(10.7–11.2)	14.4(14.0–14.8)	6.8(6.5–7.1)	16.4(16.1–16.7)	22.2(21.7–22.7)	9.5(9.1–9.8)
12-	25.6(25.1–26.1)	33.9(33.2–34.5)	15.5(15.0–16.1)	8.6(8.3–8.9)	11.2(10.7–11.6)	5.4(5.0–5.7)	17.1(16.7–17.5)	22.7(22.1–23.3)	10.2(9.7–10.7)
13-	20.9(20.1–21.7)	25.1(24.0–26.3)	15.9(14.8–16.9)	6.3(5.8–6.7)	7.7(7.0–8.4)	4.6(4.0–5.2)	14.6(13.9–15.3)	17.4(16.4–18.4)	11.3(10.8–13.1)
14-	21.2(20.2–22.3)	25.1(23.6–26.6)	16.8(15.4–18.2)	6.8(6.2–7.5)	8.6(7.6–9.5)	4.9(4.1–5.6)	14.4(13.5–15.3)	16.5(15.3–17.8)	12.0(10.8–13.1)
Total	26.8(26.7–27.0)	33.2(33.0–33.4)	19.2(19.0–19.2)	11.7(11.5–11.8)	14.5(14.3–14.7)	8.2(8.1–8.4)	15.2(15.1–15.3)	18.7(18.5–18.9)	11.0(10.8–11.1)

Obesity-related blood pressure including diastolic pressure and systolic pressure in the obesity group were higher than children without obesity, while the blood pressure of both groups was all within reference range. (Table 3) Poisson regression analysis showed that male children and female children aged 10 to 12 years had the same phenomenon. (Table 4)

**Table 3:** Obesity related blood pressure among children and adolescents in Jiangsu Province 2014–2017.

Sex	Age	Obesity		Normal		Obesity VS Normal (P value)	
		Diastolic pressure	Systolic pressure	Diastolic pressure	Systolic pressure	Diastolic pressure	Systolic pressure
Male	7	103.7±12.4	66.7±9.4	101.0±12.6	65.2±9.7	0.000	0.000
	8	106.0±11.8	67.6±8.8	102.5±12.1	66.0±9.5	0.000	0.000
	9	108.6±11.7	68.7±8.7	103.7±11.6	66.1±9.0	0.000	0.000
	10	110.7±11.7	69.9±8.7	105.5±11.6	67.1±9.1	0.000	0.000
	11	113.5±11.5	71.1±8.5	107.6±11.4	68.0±8.8	0.000	0.000
	12	116.3±11.8	71.7±8.5	110.2±11.5	68.7±8.8	0.000	0.000
	13	121.9±11.4	72.7±8.8	115.0±11.9	69.9±8.9	0.000	0.000
	14	122.3±11.6	73.7±7.9	118.4±11.5	71.5±8.8	0.000	0.000
	Total	109.4±12.6	69.1±9.0	105.4±12.4	67.0±9.3	0.000	0.000
Female	7	102.1±12.6	66.2±9.3	99.8±12.7	64.8±9.6	0.000	0.000
	8	105.1±12.0	67.4±8.5	101.1±12.0	65.5±9.2	0.000	0.000
	9	107.0±12.0	68.8±8.6	102.0±11.6	65.6±8.8	0.000	0.000
	10	110.0±12.1	69.8±9.2	104.4±11.7	66.7±8.8	0.000	0.000
	11	113.9±11.9	71.0±8.4	106.8±11.4	67.7±8.5	0.000	0.000
	12	116.1±11.2	72.1±8.5	109.0±11.2	68.9±8.7	0.000	0.000
	13	119.2±11.6	74.3±8.5	112.6±11.2	70.4±8.7	0.000	0.000
	14	120.0±11.1	76.1±8.3	113.3±10.8	71.1±8.5	0.000	0.000
	Total	107.8±13.0	68.8±9.1	104.0±12.3	66.6±9.1	0.000	0.000

**Table 4:** Poisson regression analysis of relationship between other children common disease and obesity.

Variable in All subjects	β	SE	P	RR (95%CI)
Age group one 7–9				
Diastolic BP Male	0.003	0.000	0.001	1.003(1.002–1.003)
Systolic BP Male	0.001	0.000	0.000	1.001(1.000–1.002)
Diastolic BPFemale	0.003	0.000	0.197	1.000(0.999–1.000)
Systolic BP Female	0.000	0.000	0.000	1.003(1.002–1.003)
UCVA Male	0.237	0.017	0.000	1.267(1.225–1.311)
UCVA Female	0.199	0.024	0.000	1.221(1.166–1.279)
Age group two 10–12				
Diastolic BP Male	0.003	0.000	0.000	1.003(1.002–1.003)
Systolic BP Male	0.002	0.000	0.000	1.002(1.001–1.003)
Diastolic BPFemale	0.002	0.000	0.000	1.002(1.000–1.003)
Systolic BP Female	0.003	0.000	0.000	1.003(1.003–1.004)
UCVA Male	0.118	0.020	0.000	1.125(1.082–1.169)
UCVA Female	0.054	0.031	0.082	1.056(0.993–1.123)
Age group three 13–14				
Diastolic BP Male	0.006	0.000	0.000	1.006(1.003–1.008)
Systolic BP Male	0.003	0.003	0.000	1.034(1.002–1.005)
Diastolic BPFemale	-0.001	0.006	0.870	0.999(0.988–1.010)
Systolic BP Female	0.004	0.004	0.309	1.004(0.996–1.012)
UCVA Male	0.113	0.095	0.236	1.119(0.929–1.348)
UCVA Female	0.163	0.164	0.320	1.177(0.854–1.621)

β indicates parameter estimate;

SE, standard error of estimate.

We also found that obesity group especially for male children aged from 7 to 12 years had a higher UCVA prevalence than that of normal group. However, children aged 13 to 14 years had a similar UCVA prevalence for both groups. (Table 5) For children aged 7–9 years, multiple linear regression analysis suggested obesity was associated with UCVA. (Table 4)

**Table 5:** Relationship between obesity and UCVA among children and adolescents in Jiangsu Province from 2014–2015 to 2016–2017.

Age	Obesity (95%CI)		Normal(95%)		χ^2^	P
	Male	Female	Male	Female		
7	32.4(31.1–33.7)	35.3(33.5–37.0)	26.7(26.2–27.3)	30.9(30.3–31.5)	χ^2^_male_ = 68.2 χ^2^_female_ = 22.6 χ^2^_m VS f_ = 6.9	0.000 0.000 0.009
8	37.9(36.5–39.3)	41.7(39.7–43.7)	30.4(29.9–31.0)	33.8(33.2–34.4)	χ^2^_male_ = 98.1 χ^2^_female_ = 60.7 χ^2^_m VS f_ = 9.5	0.000 0.000 0.002
9	46.3(44.9–47.7)	49.3(47.2–51.4)	39.7(39.1–40.3)	43.7(43.1–44.4)	χ^2^_male_ = 73.7 χ^2^_female_ = 24.6 χ^2^_m VS f_ = 5.3	0.000 0.000 0.021
10	52.7(51.2–54.1)	54.8(52.6–57.0)	48.5(47.9–49.1)	53.6(53.0–54.2)	χ^2^_male_ = 26.4 χ^2^_female_ = 1.0 χ^2^_m VS f_ = 2.5	0.000 0.305 0.114
11	60.5(59.0–62.0)	66.9(64.5–69.2)	57.0(56.4–57.6)	63.6(62.9–64.2)	χ^2^_male_ = 16.8 χ^2^_female_ = 7.0 χ^2^_m VS f_ = 19.7	0.000 0.008 0.000
12	67.6(65.5–69.6)	73.7(70.6–76.7)	63.5(62.7–64.2)	71.1(70.3–71.8)	χ^2^_male_ = 13.0 χ^2^_female_ = 2.5 χ^2^_m VS f_ = 10.0	0.000 0.114 0.002
13	77.8(73.8–81.8)	83.6(78.5–88.6)	73.5(72.2–74.7)	81.8(80.6–82.9)	χ^2^_male_ = 3.8 χ^2^_female_ = 0.4 χ^2^_m VS f_ = 2.9	0.052 0.513 0.091
14	83.6(79.3–87.9)	93.6(89.5–97.6)	85.8(84.6–87.0)	91.1(90.0–92.1)	χ^2^_male_ = 1.1 χ^2^_female_ = 1.0 χ^2^_m VS f_ = 8.2	0.304 0.309 0.004
Total	48.0(47.4–48.7)	50.3(49.5–51.2)	44.7(44.4–44.9)	49.7(49.4–50.0)	χ^2^_male_ = 100.0 χ^2^_female_ = 1.8 χ^2^_m VS f_ = 17.4	0.000 0.178 0.000

## Discussion

This study showed the prevalence of overweight and obesity among children aged 7 to 14 years using aged- and sex-specific cut-off points for BMI recommended by WGOC[15]. Since2014, average 108,331 children were enrolled annually in Jiangsu Province, and the prevalence of combined overweight and obesity was26.8%(33.2% for male and 19.2% for female). The prevalence of both obesity and overweight indicated there were regional and age differences. BMI value maintained stable in our three surveys. The relationship between obesity and blood pressure as well as UCVA were also explored in this study.

The prevalence of overweight and obesity was similar to other studies in China. In 2010, Song et estimated the prevalence of obesity among Chinese children aged 7 to 9 years (15.8% for boys and 8.0% for girls), 10 to 12 years (12.5% for boys and 6.5% for girls)and 13 to 15 years (8.5% for boys and 4.0% for girls), which was similar to our results[19]. Ji et noted that the prevalence of overweight among children aged 7 to 12 years was 36.8% and 24.6% in the major cities of north coast and the upper cities of south coast, respectively[20]. However, these studies were of small sample sizes and didn’t display detailed age-specific information about obesity and overweight.

Income is another factor that plays an important role in obesity[21, 22]. The income level of the residents in Jiangsu Province residents was closely associated with geographical distribution: Southern>Middle>Northern (detailed information can be referred to S2 Table from *Jiangsu Statistical Yearbook*). In the United states, the prevalence of childhood obesity was low among youths living in households in the highest income families[23]. In our study, the lowest prevalence of overweight and obesity was found in the southern region of Jiangsu Province, where the residents had the highest average income levels. However, this pattern was not seen in all families and may be affected by other factors such as education level [24]. Similar to other studies, males tend to be overweight or obesity, and reason for this phenomenon can be explained by differences in lifestyle, gene and sex behaviors and so on[9, 25–28]. The value of BMI increased by age, but the prevalence of obesity decreased by age among primary school students aged 7 to 14 years.

Childhood obesity is associated with an increased prevalence of pediatric diseases, such as type 2 diabetes, dyslipidaemia, hypertension, asthmaand so on[12, 29]. In our study, we also explored the relationships between obesity and other health issues. In obese children, their blood pressure were higher than children without obesity, while both groups were within the reference range. Several systematic reviews have confirmed that higher BMI in childhood and adolescence increases the risk of cardiovascular disease in adulthood. This finding indicated that BMI levels were positively associated with SBP and DBP levels[30, 31]. In addition, we found that obese children aged 7 to 9 years seemed to have poorer visions comparing with children without obesity, and even variations can last to 12 years of age for boys. Qi et found that the prevalence of myopia was associated with the intake of proteins such as milk, egg, beans and meat[32], which was related to high prevalence of obesity. Lim et carried out an 851-Chinese-school-children experiment in Singapore, and found that the eye axial length, which was associated with refractive error or a diagnosis of myopia, was longest in the highest quartile group of total cholesterol intake compared with the lowest group[33].

This study has several limitations. First, we only enrolled children from 41.2% districts or counties of Jiangsu Province, so the results therefore may not totally represent the prevalence of the whole province. Moreover, the observations in the study, such as the relationship between obesity and other diseases, should need for further exploration.

### Conclusion

We found that obesity/overweight prevalence differed by sex, age, and region in Jiangsu Province. In addition, obese children were closely associated with other common disease. Further studies are needed to explore the basis of biological and statistical theories.

## Supporting information

S1 Fig Distribution of study region included in Jiangsu province from 2014 to 2017. (TIF)

S1 Table Standard of screening for overweight / obesity by BMI. (DOCX)

S2 Table Income and expenditure of urban residents in northern, middle and southern of China, 2005–2014. (DOCX)
