# Supplementary figures and images for: Prevalence of overweight and obesity among primary school-aged children in Jiangsu Province, China, 2014-2017
Source: PLoS One. 2018 Aug 23;13(8):e0202681. doi: 10.1371/journal.pone.0202681 (PMC6107224; doi:10.1371/journal.pone.0202681)

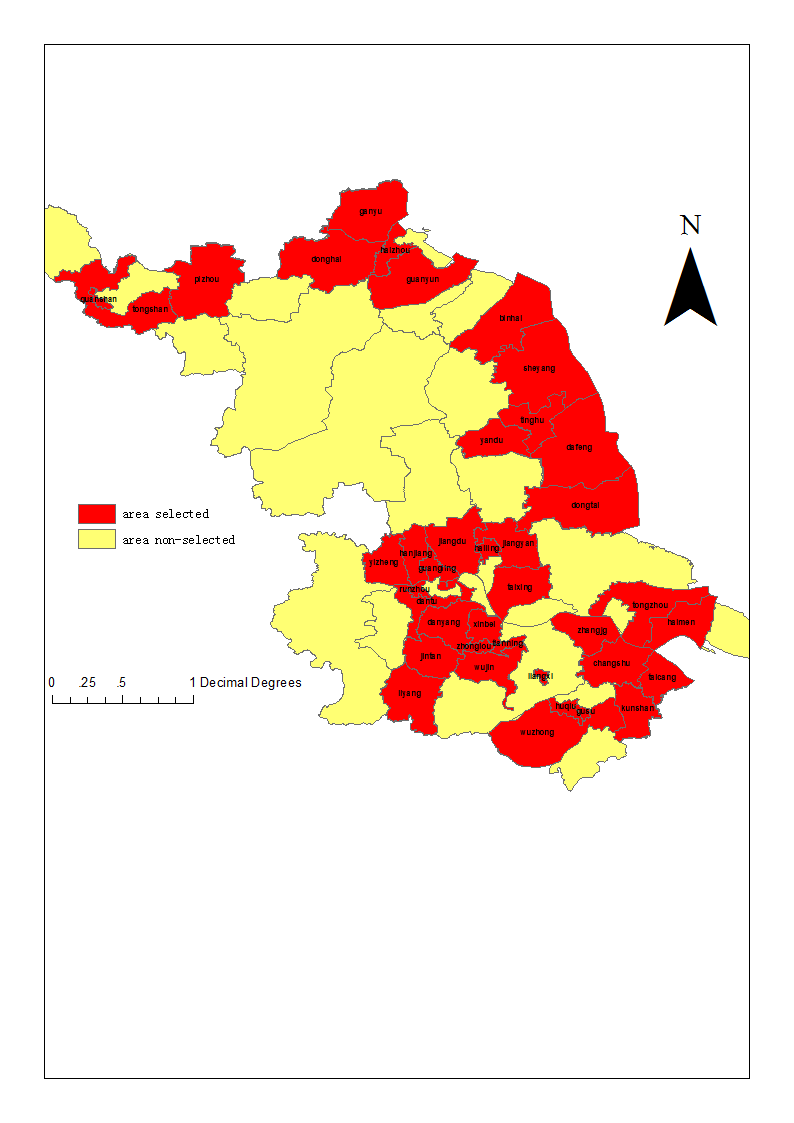

Supplement: S1 Fig — (TIF) [file pone.0202681.s001.tif]
