# Supplementary material for: Prevalence of overweight and obesity among primary school-aged children in Jiangsu Province, China, 2014-2017
Source: PLoS One. 2018 Aug 23;13(8):e0202681. doi: 10.1371/journal.pone.0202681 (PMC6107224; doi:10.1371/journal.pone.0202681)
Supplement: S1 Table — (DOCX) [file pone.0202681.s002.docx]

**S1 Table. Standard of screening for overweight / obesity by BMI**

| age | Male | | Female | |
| --- | --- | --- | --- | --- |
|  | overweight | obesity | overweight | obesity |
| 7- | 17.4 | 19.2 | 17.2 | 18.9 |
| 8- | 18.1 | 20.3 | 18.1 | 19.9 |
| 9- | 18.9 | 21.4 | 19.0 | 21.0 |
| 10- | 19.6 | 22.5 | 20.0 | 22.1 |
| 11- | 20.3 | 23.6 | 21.1 | 23.3 |
| 12- | 21.0 | 24.7 | 21.9 | 24.5 |
| 13- | 21.9 | 25.7 | 22.6 | 25.6 |
| 14- | 22.6 | 26.4 | 23.0 | 26.3 |
