# Supplementary material for: Prevalence of overweight and obesity among primary school-aged children in Jiangsu Province, China, 2014-2017
Source: PLoS One. 2018 Aug 23;13(8):e0202681. doi: 10.1371/journal.pone.0202681 (PMC6107224; doi:10.1371/journal.pone.0202681)
Supplement: S2 Table — (DOCX) [file pone.0202681.s003.docx]

**S2 Table. Income and expenditure of urban** **residents in northern, middle and southern of China,2005-2014***

| Year | Southern | | Middle | | Northern | |
| --- | --- | --- | --- | --- | --- | --- |
|  | Income(RMB) | Expenditure(RMB) | Income(RMB) | Expenditure(RMB) | Income(RMB) | Expenditure(RMB) |
| 2005 | 15083 | 10570 | 11659 | 7877 | 9116 | 7154 |
| 2010 | 27780 | 17378 | 20748 | 12988 | 14101 | 10661 |
| 2011 | 31762 | 20244 | 24052 | 15042 | 16020 | 12353 |
| 2012 | 35827 | 22786 | 27095 | 17103 | 18415 | 13837 |
| 2013 | 39224 | 24728 | 29706 | 19063 | 22933 | 15851 |
| 2014 | 42753 | 26487 | 31969 | 20336 | 24177 | 14956 |

*: Data were acquired from *Jiangsu Statistical Yearbook*
